# Career options after Bachelor of Ayurvedic Medicine and Surgery

**DOI:** 10.4103/0974-7788.72495

**Published:** 2010

**Authors:** Urmila A. Pitkar

**Affiliations:** *MD (Ayu), PhD, E-mail: uapitkar@gmail.com*

In the past, there was only one option available for freshly graduated doctors (after passing BAMS), and that was to start one’s own practice. However, today there are many promising opportunities available for BAMS graduates. The areas which are available for a BAMS graduate to build a career successfully can be broadly classified into the following five categories:

- Clinical practice
- Academic
- Research
- Management and administration
- Drug manufacturing

The present article discusses some of these options in detail. While selecting a career, a student should try to match his or her own interest, basic qualities as well as specific qualities, which a specific field may demand (e.g., a student planning to pursue a career in management must have good communication skill), and the resources available to him/her with the requirement of the career option.

## CLINICAL PRACTICE

A student entering Ayurvedic practice cannot avoid the detailed study of Ayurvedic *Samhitas*. The student should also be well aware about modern medical science and interpretation of modern diagnostic tests. Practical experience is equally required for becoming a successful practitioner. It is advisable that a student works with senior practitioners for at least 1–2 years before setting up his own practice. Along with regular practice, a student can also opt for specialized practice after acquiring proper knowledge, e.g., therapy of skin diseases, spinal disorders, ophthalmologic conditions, ano-rectal diseases, etc., where Ayurveda has a special role to play today.

In India, there are various traditions of Ayurvedic practice like *Panchbhautik Chikitsa, Nadi Pariksha, Dhatu Chikitsa*, etc. Students can learn such traditions and follow them in their practice.

Students can add value to their practice by combining various therapies such as *Panchakarma*, Yoga, Ayurvedic dietetics, Counseling, Physiotherapy, etc., by doing additional diploma/certificate courses available.

A postgraduate diploma in Emergency Medicine, which is a short course of 6 months conducted in Modern Tertiary Healthcare Hospitals (e.g., Hinduja Hospital in Mumbai or Ruby Clinic in Pune) can help give value addition to the clinical practice of a BAMS student.

## ACADEMICS

When a student plans to pursue a career in academics, it is necessary to do post graduation. Students, who cannot get opportunity for MD, can go for post graduate diplomas available in various subjects such as *Panchakarma, Balaroga*, etc.

In Maharashtra, almost 16 subjects are available for MD in 15 government colleges and 10 private colleges. In India, Institutions like BHU, Gujarat Ayurved University (Jamnagar), National Institute of Ayurveda, Jaipur, Kerala and Karnataka University have post graduation facility. The student has to appear for an entrance exam for securing admission for MD. More information about these institutions is available at the website of CCIM. MPhil available at Pune University is also an option.

Anyone who has 10 years of clinical experience or 5 years of teaching experience is eligible for PhD. Almost all the above institutions have facilities for PhD. Ayurved students can even do PhD in non-Ayurvedic subjects like analytical chemistry, biochemistry, etc.

After post graduation, students can join government or private colleges as demonstrators or lecturers.

There is a large space in private sector for the teaching profession. Private tuition classes for BAMS students, tuition classes for entrance exams, conducting workshops to teach particular skills, i.e., *Kshrasutra, Agnikarma, Panchakarma*, etc., are good options.

Recently, there are MSc programs made available in various science disciplines like Anatomy/Physiology/Microbiology for BAMS students, which are offered by many universities in Karnataka/Tamil Nadu/Maharashtra (University of Madras, Kasturba Medical College, Manipal, Sri Ramachandra Medical college, M. S. Ramaiah Medical College, Jawaharlal Institute of Post Graduate Medical Education and Research, etc.)

## RESEARCH

Apart from MD and PhD courses, there are other good courses useful for a career in research such as MSc or PG Diploma in Clinical Research. These courses are available at various institutions and universities throughout the country. Cranfield University (UK), through its branches in India, also offers a masters degree in clinical research. The duration of such studies varies from 6 months to 2 years. The course modules are extensive and include various aspects of clinical research with more focus on data analysis and management. After completion of this course, students can get job as Clinical Research Associate in research unit of pharmaceutical companies.

MSc (Biotechnology/Bioinformatics/Health Sciences) is a very challenging course and is also available for BAMS students at some universities. This is a 2-year, full time course.

Students who are interested in fieldwork as well as research can go for this course which has promising opportunities in future. Some courses useful for research careers are short course on statistics and epidemiology (Christian Medical College, Vellore), and Clinical Toxicology (Kalina).

In the research field, a student can join as Jr. Research Fellow on research projects which are conducted by various institutions/colleges/university departments which are financially supported by ICMR, CSIR, CCRAS, DST, etc. On getting experience, they can get Sr. Research Fellowship and this work can lead to a PhD.

## MANAGEMENT

For students who are not interested in clinical practice but want to have a career related to the medical field, there are ample choices. MPH (Masters in Public Health), MHA (Masters in Health Administration) and MBA (Hospital and Healthcare management) are in great demand.

Other than this, courses like Sports Medicine, Disaster Management, Industrial Management, Preventive and Promotive healthcare, Masters in Personnel Management are also good options.

UPSC examinations and state level administration examinations are conducted every year. BAMS students are eligible to appear for these examinations and enter in the government services as administrator.

## DRUG MANUFACTURING

In the manufacturing sector, production of Ayurvedic medicines is a booming business. Apart from actual manufacturing, other allied aspects such as cultivation of medicinal plants, trading raw materials in the form of powder, extracts, oils, etc., are also in great demand. Not only medicines, but also Ayurvedic cosmetics and food products have equally big market. It is the need of time that many Ayurvedic graduates should come in this field and use their knowledge and skill.

Courses such as BPharm (Ayurved) in Jamnagar and BHU, MSc (Pharmaceutical Medicine), MBA (Medicinal Marketing) (MUHS, Nasik), Diploma in Herbal Medicinal Manufacturing (Pune University and IPER Pune) are also available.

## MISCELLANEOUS

There is great potential in manufacturing of equipments required for Ayurvedic treatments like *Panchakarma*.

Medical transcription, medical tourism, medical event management, medical journalism, medical photography and documentation are also fields with a bright future. BAMS graduates can complete LLB and work as legal medical advisor.

**Useful links:**

- http://www.madras-university.in/community.php?comm_id=122
- http://www.tmu.ac.in/course-medical-msc.html
- http://www.cranfield.ac.uk/health/postgraduatestudy/taughtcourses/msc%20clinical%20research%20india/index.html
- http://www.amity.edu/aib/programs/postgraduate/mba/about_course.asp
- http://www.osmania.ac.in/BioInformatics2009.pdf
- http://www.manipal.edu/Institutions/Medicine/KMCManipal/Courses/PostGraduate/Pages/MScMedical.aspx
- http://www.ayurveduniversity.edu.in/cou_no9.php
- http://www.tmv.edu.in/ayu_mas_mad.asp
- http://www.tmv.edu.in/ayu_mas_mbaap.asp
- http://ccimindia.org/regulations_pg_diploma_course.html
- http://www.imsbhu.nic.in/ayurveda/ayurcourse.htm
- http://www.bharatividyapeeth.edu/Campuses/Pune/College-of-Ayurved,-Pune/default.aspx
- http://ceast.puchd.ac.in/mph/courses.php
- http://www.apolloiha.ac.in/faq.html
- http://www.sriramachandra.edu.in/
- http://ccimindia.org/colleges_status_ayurveda_2010-11.php
- http://www.catalystclinicalservices.com/Pages/PDCR.htm

Courtesy: Dr. Kishor Patwardhan Assistant Professor, Department of Kriya Sharir, Faculty of Ayurveda, Institute of Medical Sciences, Banaras Hindu University, Varanasi E-mail: patwardhan.kishor@gmail.com

After doing WAVE (World-class Academy for Vocational Excellence), a 3-month short course conducted by Maharashtra Knowledge Corporation Limited (MKCL) about retail and logistics, one can start his own business to fulfill various needs of hospitals or Ambulance service.

The present article is an attempt to explore possible fields where an Ayurveda student can satisfy his professional as well as monitory needs. This article, however, does not cover all required details for all these opportunities. Personal interactions and discussions with students in various ways, e.g., career counseling, campus interviews, job fairs, student apprenticeship through an academia–industry joint initiative can help a student in better way.

